# Heparan sulfate hexasaccharide selectively inhibits cancer stem cells self-renewal by activating p38 MAP kinase

**DOI:** 10.18632/oncotarget.12358

**Published:** 2016-09-30

**Authors:** Nirmita J. Patel, Chetna Sharon, Somesh Baranwal, Rio S. Boothello, Umesh R. Desai, Bhaumik B. Patel

**Affiliations:** ^1^ Hunter Holmes McGuire VA Medical Center, Richmond, VA 23249; ^2^ Division of Hematology, Oncology, and Palliative Care, Department of Internal Medicine and Massey Cancer Center, Virginia Commonwealth University, Richmond, VA 23298; ^3^ Department of Medicinal Chemistry and Institute for Structural Biology, Drug Discovery and Development, Virginia Commonwealth University, Richmond, VA 23219

**Keywords:** cancer stem cells, colorectal cancer, glycosaminoglycans, p38 mitogen activated protein kinase, heparan sulfate

## Abstract

Heparan sulfate (HS) plays a role in the majority of essential hallmarks of cancer, yet its ability to modulate self-renewal, especially of cancer stem cells (CSCs), remains unknown. We have discovered that a non-anticoagulant HS hexasaccharide (HS06) sequence, but not other shorter or longer sequences, selectively inhibited CSC self-renewal and induced apoptosis in colorectal, pancreatic, and breast CSCs suggesting a very general phenomenon. HS06 inhibition of CSCs relied upon early and sustained activation of p38α/β mitogen activated protein kinase (MAPK) but not other MAPKs family members i.e. ERK and JNK. In contrast, polymeric HS induced exactly opposite changes in MAPK activation and failed to inhibit CSCs. In fact, TCF4 signaling, a critical regulator of CSC self-renewal, was inhibited by HS06 in a p38 activation dependent fashion. In conclusion, HS06 selectively inhibits CSCs self-renewal by causing isoform specific activation of p38MAPK to inhibit TCF4 signaling. These observations on chain length-induced specificity carry major mechanistic implications with regard to HS in cancer biology, while also presenting a novel paradigm for developing novel anti-CSC hexasaccharides that prevent cancer relapse.

## STATEMENT OF SIGNIFICANCE

Heparan sulfate (HS) of specific length, i.e., hexasaccharide (HS06), but not longer or shorter sequences, selectively inhibit cancer stem cells (CSCs) through isoform specific activation of p38 mitogen-activated protein kinase. These findings will have major implication for developing chemical probes to decipher complex signaling events that govern cancer stem cells. Additionally, there are direct implications for designing glycosaminoglycan based cancer therapies to selectively target CSCs that escape killing by traditional chemotherapy threatening cancer relapse.

## INTRODUCTION

Complete cure of cancer is almost never achieved because all current anti-cancer agents primarily target the bulk of tumor, and not the small population of cancer stem cells (CSCs) that seed every tumor [1–5]. CSCs are endowed with the ability to self-renew, invade through matrix and differentiate to bulk cells; properties collectively referred to as the CSC phenotype. CSCs are thought to be highly tumorigenic and resistant to conventional chemotherapy and/or radiation [1, 6], which helps them withstand even intensive anti-cancer regimen. CSCs may also lay dormant before incidental activation, which may explain disease relapse [2, 7]. Despite the challenges, the CSC paradigm presents a major opportunity for developing novel agents that offer the possibility of permanent disease cure. In fact, several approaches have been presented with the goal of identifying anti-CSC agents [1, 8–10] and several clinical trials are currently in progress.

Many of the hallmarks of cancer, especially sustained proliferation, evasion of suppression, angiogenesis, and metastasis [11], are modulated to a significant extent by glycosaminoglycans (GAGs) and proteoglycans (PGs) [12, 13]. These effects are mediated through interactions with a number of proteins, mostly on the cell surface, to modulate intracellular signal transduction [13, 14]. Although GAG interactions with cell surface receptor(s) are likely to be pleotropic, the sum product of signals transduced result in alteration of protein kinase activation, especially PKB, PKC and/or mitogen-activated protein kinases (MAPK) [15–17]. In fact, many of these pathways play critical role in regulation of the hallmarks of cancer described above [15].

The template-less and spatiotemporal biosynthesis of HS, which is further fine-tuned by processing enzymes such as sulfatases and heparanase, generates massive compositional, configurational and conformational diversity. Nature appears to exploit this diversity for engineering myriad physiological functions [18], the majority of which remain poorly defined. A growing paradigm is that distinct HS sequences are involved in modulating these functions. Although no sequence other than heparin pentasaccharide Arixtra (HS05) has reached the clinic to date, a large number proteins appear to bind to GAGs with high specificity [19], which when coupled with the phenomenal diversity of the GAGome, raise the promise of realizing multiple GAG-based drugs.

We reasoned that this promise could help selectively target CSCs *versus* bulk cancer cells. Although many of the same HS-binding proteins function in both bulk cancer cells and CSCs, we hypothesized that there may be differential activation of HS-binding proteins between the two cancer cell types. In fact, our earlier work with a select group of GAG mimetics alluded to the possibility of selective targeting of CSCs by a distinct HS sequence [10]. Using the orthogonal, tandem screen, developed earlier by us [10, 20], we have now discovered that CSCs can be selectively inhibited by a chain length-specific sequence of HS. This was found to be a multi CSC (colon, pancreas, breast) phenomenon and likely to be even more general. Interestingly, the HS hexasaccharide sequence (HS06) produced isoform specific, sustained activation of p38 MAPK but inhibition of ERK, another MAPK family member. In contrast, longer chains of HS displayed p38 inhibition and ERK activation revealing for the first time a new dimension of complexity in understanding the ‘HS-elephant’ by ‘six blind men’ [21].

## RESULTS

### Chain length dependence of GAG inhibition of CSCs

Recently we developed a simple, orthogonal, tandem screening strategy to identify agents that selectively target CSCs [10, 20]. In this strategy, agents that inhibit the growth of a cancer cell line under spheroid conditions (which enrich CSCs), but not under monolayer conditions (which deplete CSCs) are identified in the first screen (primary culture, 1°) and then assessed for inhibition of secondary (2°) and tertiary (3°) spheroid growth in the absence of any added agent. The 2° and 3° screens are particularly important because it helps distinguish putative inhibitors that might target early progenitors, and hence inhibit 1° growth, but might not affect CSC self-renewal.

**Figure 1:**
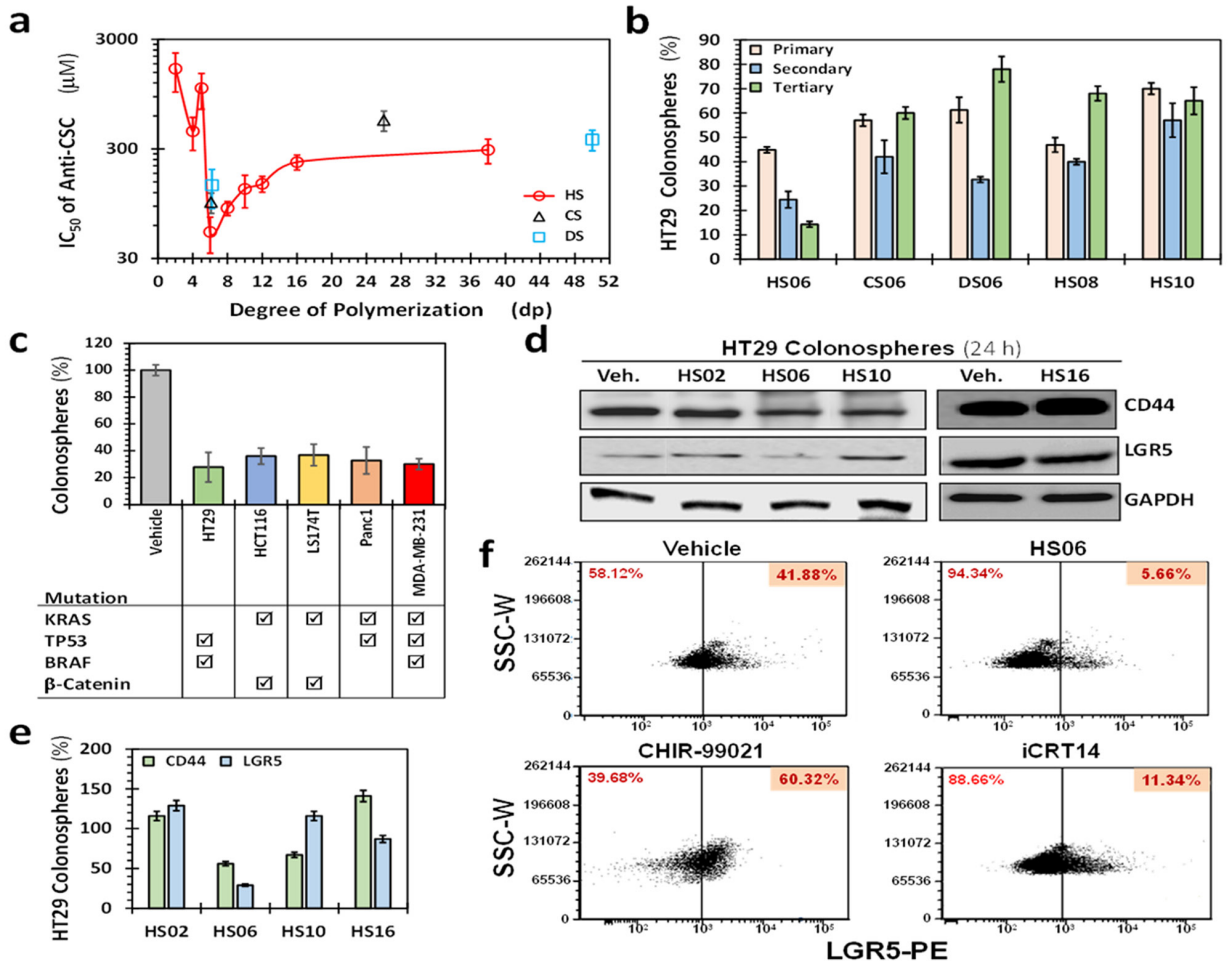
HS06 selectively inhibits CSCs growth **a**. Scatter plot showing *IC*_50_ of primary (1°) spheroid growth of HT29 cells as a function of the degree of polymerization of sulfated GAGs. Error bars represent ±1 SD (n ≥ 3). **b**. Results of the secondary (2°) and tertiary (3°) spheroid growth in the absence of further GAG treatment in HT29 cells (p53 mutant, K-RAS wild type, microsatellite stable) for most promising agents (*IC*_50_ < 100 μM) identified in the 1° screen. HS06 was the only GAG that satisfied the predefined cutoff of >50% inhibition in both 2° and 3° spheroids growth. Data presented refers to percent of the vehicle control. Error bars represent ±1 SEM. **c**. Comparative effect of 100 μM HS06 on 1° spheroid formation in colon (HCT116, HT29, LS174T), pancreas (Panc1), and breast (MDA-MB-231) cancer cells. Check boxes indicate the mutational status of each of the cancer cell line (from http://cancer.sanger.ac.uk/cosmic). **d**. Western blot analyses showing expression of markers CD44 and LGR5 on HT29 CSCs in the presence of 100 μM of HS02, HS06, or HS10. Only HS06 showed robust (>50%) inhibition of these markers. **e**. Bar graph representing relative expression of CSC markers normalized to vehicle control. **f**. Flow cytometric evaluation of LGR5 following treatment with 100 μM HS06 or 100 nM CHIR99021 or 40 nM iCRT 14, a stimulator and an inhibitor of LGR5 expression, respectively.

We applied this dual screening strategy to a library of sulfated GAGs, i.e., HS and chondroitin sulfate (CS), of varying chain lengths and constitution. The rationale for choosing this library was that growth factor/chemokine signaling is known to be variably modulated by chains of different length [22, 23]. None of the GAGs studied caused growth inhibition of colorectal HT29 cancer cells in monolayer condition above a threshold of 50% at a dose less than 300 μM (Supplementary Figure S1). In contrast, several GAG sequences inhibited HT29 spheroid growth (Supplementary Figure S2). Whereas HS02, HS04 and HS05 were essentially ineffective, HS06 through HS12 (HS06>HS08>HS10>HS12) caused significant inhibition of 1° spheroid formation. However, chains larger than HS12 demonstrated significant loss of inhibitory potential (Figure 1a). CS and DS also displayed a qualitatively similar profile, yet, there was considerable difference between the three agents of the same chain length (HS06>CS06>DS06) in their ability to inhibit 1° spheroid growth (Figure 1a). Intriguingly, anticoagulant activity of HSGAGs inversely correlated with their anti-CSC IC_50_ values (Supplementary Table S1), i.e. HS06, the most potent anti-CSC GAG sequence is also naturally non-anticoagulant, a desirable property for an anti-cancer agent.

We selected HS06, CS06, DS06, HS08 and HS10 for 2° and 3° spheroid screening of HT29 and HCT116 self-renewal in the absence of further treatment. Of note, the two cell lines, HT29 (p53 mutant, K-RAS wild type, microsatellite stable) and HCT116 (p53 wild-type K-RAS mutant, microsatellite instable), differ significantly in their cancer-specific genetic background [24]. Although some inhibition of 2° and 3° spheroid formation was observed with most oligosaccharides, only HS06 produced robust (>66%), progressive, and sustained inhibition of self-renewal in both cell lines (Figure 1b, Supplementary Figure S3). We also observed a similar phenomenon in other cancer cells tested, including colon (LS174T), pancreatic (Panc-1), and breast (MDA-MB-231) (Figure 1c), suggesting that these effects may be generally true for majority of CSCs.

To confirm that these phenotypic effects are indeed arising from effects on CSCs, we studied expression of CD44 and LGR5, two typical molecular markers associated with CSCs [25]. Only HS06 caused a robust decrease in both CD44 and LGR5 (~45-75%, Figures 1d, 1e). Additionally, flow-cytometric analyses for LGR5 showed a robust (>75%) reduction in LGR5 (hi) cells following treatment with HS06 in comparison to vehicle-treated controls (Figure 1f). In contrast, at equimolar concentrations, ineffective GAGs, e.g., HS02, HS10, and HS16, induced minimal to modest inhibition in expression of these CSCs markers (Figures 1d, 1e). In combination, the results demonstrate that optimal type and chain length of GAG is critical for selective inhibition of CSC growth. Advantageously, this phenomenon holds true for several different types of CSCs.

### HS06 inhibits self-renewal and activates apoptosis in CSCs

**Figure 2:**
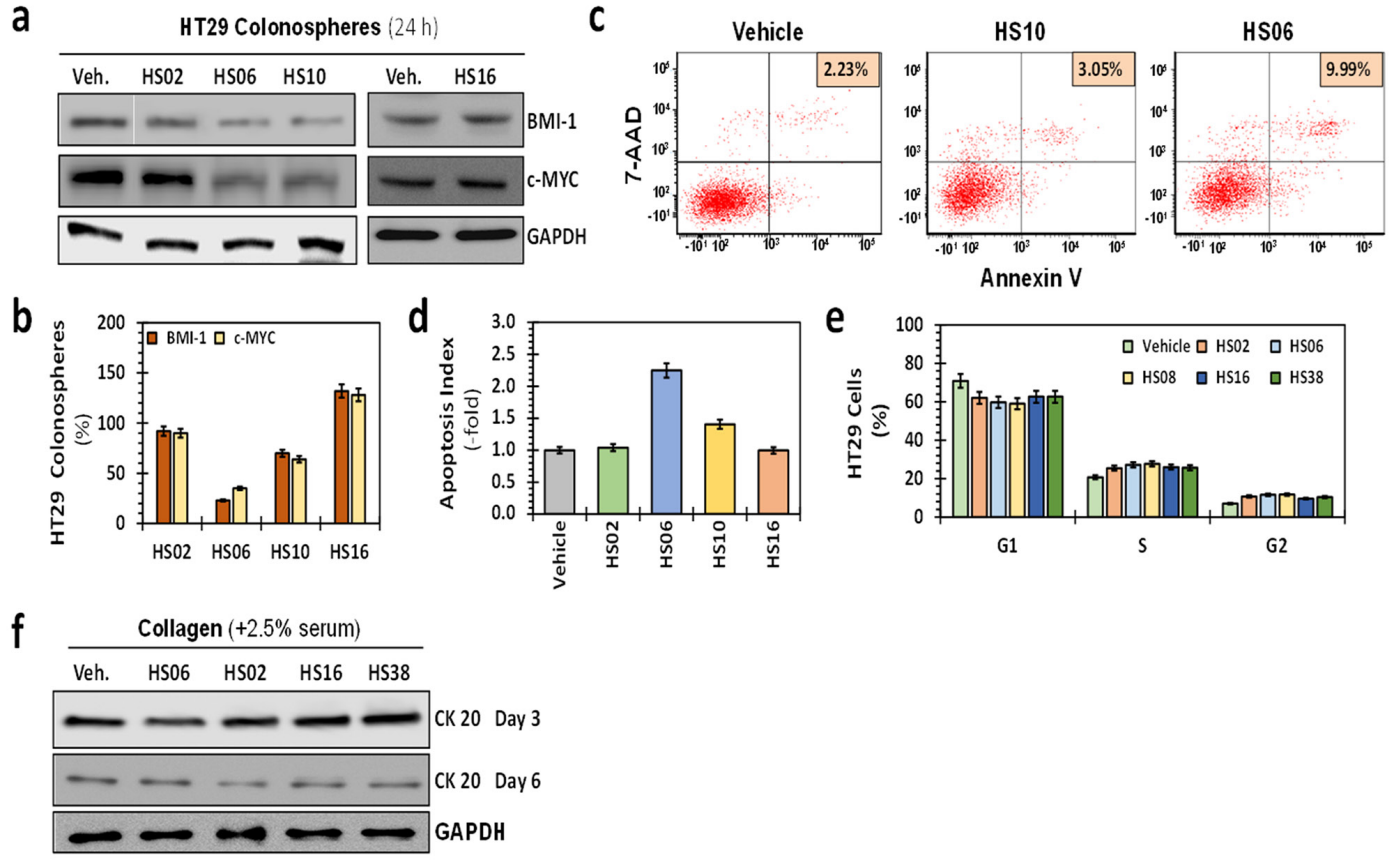
Effects of HSGAGs on broad cellular processes of HT29 CSCs **a**. Western blot analyses of expression of self-renewal factors BMI-1 and c-MYC in primary HT29 spheroids in the presence of 100 μM sulfated GAGs. **b**. Bar graph representing quantitative changes in the relative expression of CSC markers and self-renewal factors. Whereas HS02, HS10, and HS16 produced only a modest (<50%) effect on the expression of these markers, HS06 induced robust 60–80% inhibition. **c**. Flow cytometry was used to assess apoptosis in which Annexin V expression in HT29 spheroids was identified by 7-amino-actinomycin-D (7-AAD) labeling. The proportion of CSCs in each sample is listed on top right. **d**. Bar graph of relative apoptotic index ({apoptotic cells (reference) / total cells (reference)}) using fluorescence microscopy to assess morphological changes induced by 100 μM GAGs at 24 hrs post-treatment (acridine orange–ethidium bromide (AO-EB) staining). Apoptosis induction using two different methods shows robust induction with HS06, modest increase with HS10, and no change with inactive molecules HS02 or HS16 compared to vehicle-treated control at 24 hrs post-treatment with GAGs. Error bars represent ±1 SEM. **e**. Relative cell cycle distribution at 24 hrs following GAG treatment was assessed by fixing cells in 70% EtOH or 100% MeOH and incubating for 30 min at -20 °C. Error bars represent ±1 SEM. **f**. Western blot showing expression of colonic differentiation marker CK20 in HT29 colonospheres at day 3 and 6. Differentiation was induced by growing the cells on collagen matrix in the presence of 2.5% serum.

To gain insight into the mechanism of selective CSC targeting by HS06, we examined its effect on broad cellular processes in spheroid cells. Consistent with its effect on 2° and 3° spheroids, HS06 caused a significant inhibition of self-renewal factors BMI-1 and c-MYC (Figures 2a, 2b). In comparison, inactive GAGs including HS02, HS10 and HS16 induced no such changes. Further, HS06 enhanced apoptosis of HT29 spheroids ~2.5-fold in comparison to vehicle-treated controls in two different assays, namely acridine orange/ethidium bromide (AO/EB) staining and annexin V labeling (Figures 2c, 2d). In contrast, HS02 and HS16 demonstrated no such effects, while HS10 caused a modest (~1.4-fold) increase in apoptotic cells. We also found essentially no influence of HS06 on cell cycle and cellular differentiation (Figures 2e, 2f). These findings suggest that HS06 inhibits CSCs through attenuation of self-renewal as well as enhancement of apoptosis.

### HS chain length dependent regulation of mitogen activated protein kinases

**Figure 3:**
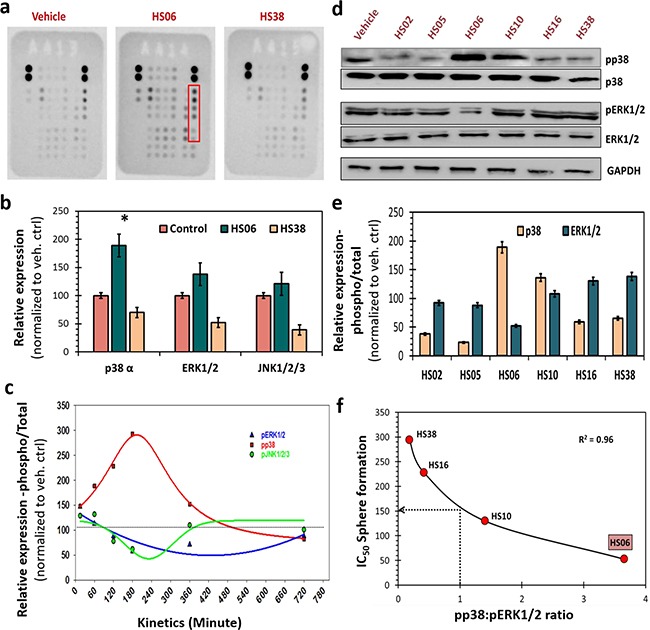
HSGAGs modulate p38 and ERK1/2 MAPKs in CSCs **a**. Human phospho-kinase array (R&D) identified increased levels of phosphorylated-form of three related proteins p38α, ERK1/2 and JNK1/2/3 (red box) at ~15 minutes following treatment with HS06, but not with HS38 (both 100μM) or vehicle. **b**. Bar graph representation of relative densitometry values of phosphorylated MAPKs compared to vehicle control. **c**. Kinetics of MAPK activation (15min → 720min) - shows early and sustained activation of p38MAPK. Whereas ERK1/2 and JNK1/2/3 showed very brief activation followed by basal activation (JNK) or even modest inhibition (ERK) with HS06. **d**. Western-blot analyses of expression of phosphorylated-MAPKs (pp38, pERK1/2) in spheroid conditions following treatment with varying chain length HSGAGs (HS02→HS38) at 100μM concentration revealed specific chain length dependent activation of p38 and/or ERK1/2. Specifically, HS oligosaccharide (HS06>HS10) showed p38 activation and ERK1/2 inhibition; whereas HS polymers (HS16 and HS38) as well as those smaller than HS06 showed the opposite effect suggesting unique chain length based structure-activity-relationship. **e**. Bar graph of relative densitometry values of phosphorylated over total MAPK. **f**. Graphical representation of strong inverse correlation (r^2^ = 0.96) between pp38:pERK1/2 ratio and anti-CSC potency of HSGAGs. HSGAGs showing pp38:pERK1/2 >1 have lower IC50 (<150 μM) for spheroid growth than those with the reverse pp38:pERK1/2 ratio. GAPDH was used as loading control. Error bars represent ±1 SEM. * p-value < 0.005.

Although the precise mechanism(s) of CSC growth and self-renewal are poorly understood, several key cytoplasmic kinases, especially involving growth factor, morphogen and/or cytokine signaling, are known to contribute [26]. We performed a human phospho-kinase antibody array (R&D systems, Minneapolis, MN) to detect site-specific phosphorylation of 43 kinases, representative mediators of key cancer and/or stem cell signaling, in HT29 spheroids treated with vehicle, HS06 and HS38. Only a handful of kinases showed a significant change in phosphorylation following HS06 treatment, but not HS38 treatment, compared to vehicle treatment (Figure 3a, Supplementary Table S2). Of these, three related kinases, p38, ERK1/2 and JNK1/2/3, belong to the mitogen-activated protein kinase (MAPK) family pinpointing MAPK activation as a potential mechanism of action of HS06 (Figure 3a, 3b). Time course analysis of HS06 treatment revealed that p38 was activated early (15 min) reaching a maximum at ~3 h and returning to baseline by ~10 h in HT29 spheroids (Figure 3c). On the other hand, HS06 caused only a brief activation of ERK1/2 (15 min), which was followed by significant inhibition in ERK1/2 activation even as p38 activation reached a peak (Figure 3c). This effectively implied a significantly enhanced pp38:pERK ratio (Figures 3c–3e). At the same time, no meaningful changes in JNK, another MAPK member, were observed. Intriguingly, HS16 and HS38 caused reciprocal changes in p38 and ERK1/2 activation, which correlated with their anti-CSC effects (Figures 3d–3f). Specifically, each HS oligosaccharide with significant anti-CSC effects (i.e., IC_50_ ≤150 μM) caused significant p38 activation compared to ERK1/2 (i.e., pp38:pERK ratio >1). On the other hand, HS devoid of significant anti-CSC effects, e.g., HS02, HS05, HS16 and HS38, induced the opposite changes (i.e., pp38:pERK1/2 ratio <1) (Figure 3f). Hence, HS endowed with potent anti-CSC effects appear to cause sustained activation of p38 MAPK to regulate CSC growth and self-renewal.

### Activation of p38 MAPK is critical for HS06's inhibition of CSC self-renewal

**Figure 4:**
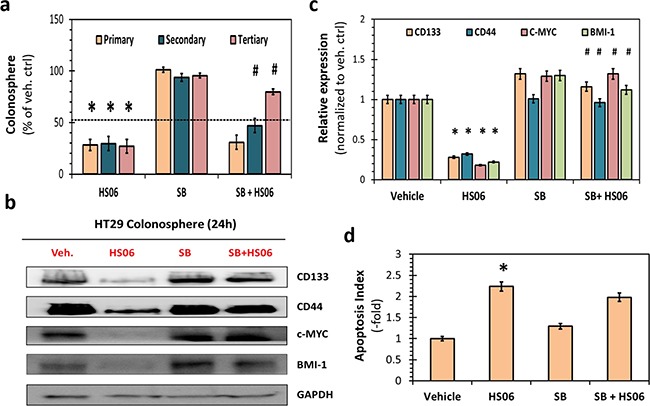
Activation of p38MAPK is critical for HS06's effect on CSCs growth and self- renewal **a**. HS06 (100 μM) induced inhibition of HT29 CSC self-renewal (i.e. 3° sphere formation) was almost completely reversed by pre-treatment with SB203580 (SB) (5μM), a pharmacologic inhibitor of p38α/β. Data is presented as percent of vehicle control. **b**. Western blot analysis and **c**. the corresponding bar graph of relative densitometry values normalized to control shows complete reversal of HS06's attenuation of CSC markers (CD133, CD44) and self-renewal factors (c-MYC, BMI-1) levels by SB. **d**. Bar graph representation of relative apoptosis index measured following AO/EB labeling of the cells. Apoptosis induction seen with HS06 is not reversed with pre-treatment with SB. GAPDH was used as loading control. Error bars represent ±1 SEM. * p-value < 0.005 compared to vehicle control. # p-value < 0.005 compared to HS06.

To further confirm the role of p38 MAPK activation in HS06 inhibition of CSCs, we examined the effects of pre-treating HT29 spheroids with SB203580, a selective pharmacological inhibitor of p38α/β [27]. While SB203580 itself caused minimal changes in CSCs growth and self-renewal, it induced partial reversal of inhibition of primary sphere formation by HS06 (Figure 4a). This effect was even better expressed for 3º spheroids, wherein SB203580 caused essentially complete reversal of HS06-mediated inhibition of CSC self-renewal (Figure 4a). This phenotypic observation was also supported at a molecular level. A near complete reversal of inhibition of CSCs markers (CD44 and CD133) and self-renewal factor (c-MYC, BMI-1) by HS06 was observed when p38 activation was abrogated by SB203580 (Figure 4b, 4c). However, pre-treatment with SB203580 did not result in significant reversal of apoptosis induction by HS06 in HT29 spheroids (Figure 4d). These results further support the conclusion that effects of HS06 on CSC self-renewal, but not apoptosis, are likely mediated by p38 MAPK activation.

### HS06 exhibits isoform-specific activation of p38 MAPK to modulate CSC self-renewal

**Figure 5:**
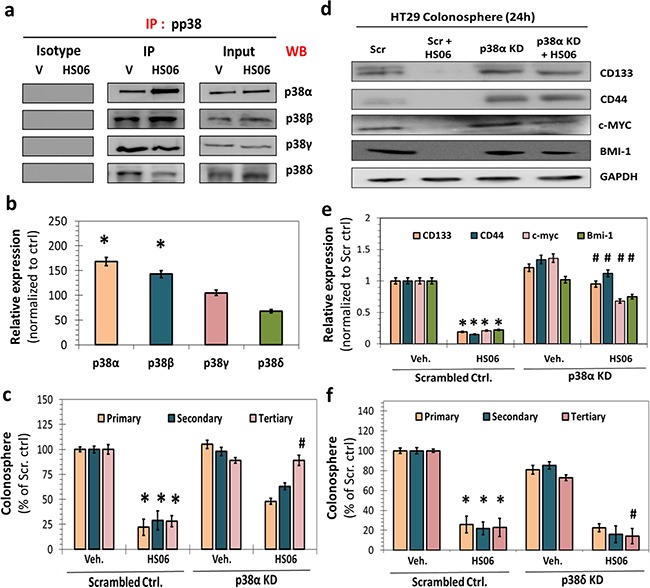
Isoform specific activation of p38MAPK is critical for HS06's effects on CSCs growth and self-renewal **a**. Immuno-precipitation (IP) with anti-pp38 followed by western blotting (WB) with isoform specific anti-p38 antibody and **b**. the corresponding bar graph representation of relative expression shows activation of α and β isoforms, inhibition of δ isoform, and no significant change in γ isoform upon HS06 treatment (100 μM @ 3hr) in HT29 cells. **c**. Effect of HS06's inhibition on HT29 CSC self-renewal (3° spheroids) is completely attenuated in the presence of p38α siRNA (KD) compared to scrambled controls. **d**. Western blot analysis and **e**. the corresponding bar graph representation of relative expression of CSC markers (CD133, CD44) and self-renewal factors (c-MYC, BMI-1) shows complete reversal HS06's inhibition in the presence of p38α siRNA at 24 hr. GAPDH was used as loading control. **f**. Effect of HS06 on HT29 CSCs’ self-renewal (3° spheroid formation) is enhanced in the presence of p38δ siRNA compared to scrambled controls. GAPDH was used as loading control. Error bars represent ±1 SEM. * p-value < 0.005 compared to vehicle control. # p-value < 0.005 compared to HS06.

As a stress-activated MAPK, p38 is responsive to various cellular stress and/or cytokines and mitogens. It mediates broad range of physiological as well as pathological processes ranging from development to cancer, which depend on the context of activation as well as isoform-specific engagement of substrates [28, 29]. To determine HS06's effect on relative activation of 4 known p38 MAPKs (α, β, γ and δ), we performed immunoprecipitation with anti-pp38 antibody followed by Western blotting with isoform-specific p38 antibodies following vehicle or HS06 treatment in HT29 spheroids. HS06 treatment resulted in activation of α and β isoforms (α > β), inhibition of the δ isoform, and no discernible effect on the γ isoform (Figure 5a, 5b). This indicates isoform-specific activation of p38 MAPK by HS06 (Figures 5a, 5b). To further clarify the role of p38 isoforms, we examined 1°, 2° and 3° HT29 spheroid growth following genetic knockdown of p38α and p38δ after respective treatments. Inhibition of p38α levels produced effects nearly identical to SB203580, a specific inhibitor of p38α/β [27], on the spheroid growth suggesting positive role of p38α activation in mediating anti-CSC effects of HS06 (Figure 5c, Supplementary Figure S4). Moreover, there was near complete reversal of CSCs markers and self-renewal factors by HS06 following p38α knockdown (Figure 5d, 5e). On the other hand, suppression of p38δ levels resulted in augmentation of HS06's inhibition of 3° spheroids suggesting that p38δ inhibition by HS06 contributes to its anti-CSCs effects (Figure 5f). Overall, the above results point to a highly specific modulation of certain p38 MAPK isoforms which appear to play opposing roles in the regulation of CSCs self-renewal by HS06.

### HS06 selectively induces p38 MAPK in CSCs

**Figure 6:**
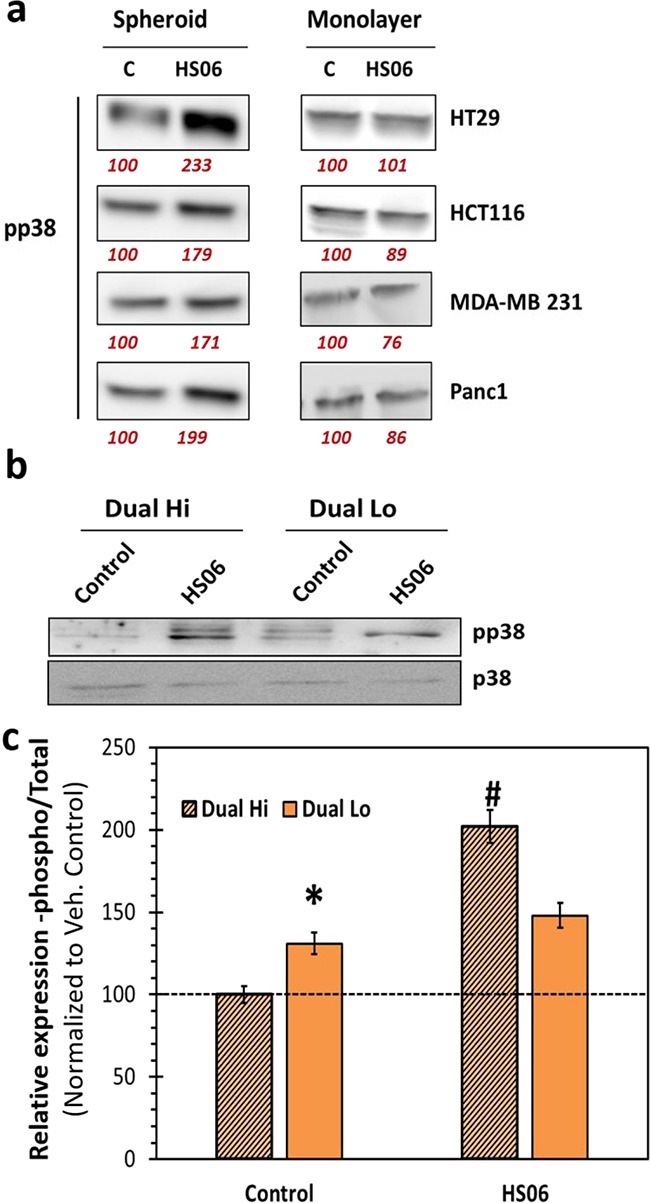
Effects of HS06 on activation of p38MAPK is selective in CSCs **a**. Western blot analysis of pp38 levels in colon (HT29, HCT116), breast (MDA-MD-231), and pancreatic (Panc1) spheroids (CSCs enriched) vs. monolayer (CSCs poor) cells shows selective p38 activation in CSCs. **b**. Western blotting and **c**. the corresponding bar graph representation of relative expression of pp38 levels in Dual hi (CD133+/CXCR4+, also CSCs) and Dual lo (CD133-/CXCR4-) HT29 cells. Numbers in red under the blot represent relative densitometry values. Error bars represent ±1 SEM. * p-value < 0.005 compared to Dual hi; # p-value < 0.001 compared to Dual hi.

One of the most intriguing findings in our screen for anti-CSC GAG sequences was selective inhibition of CSCs by HS06. Hence, we hypothesized that HS06 might induce anti-CSC signaling selectively in CSCs. Indeed, HS06 caused a robust 1.7 to 2.3-fold increase in phosphorylated form of p38 at 3 h in various colorectal (HCT116 & HT29), pancreatic (Panc-1), and breast (MDA-MB-231) cancer spheroids, but not in their monolayer counterparts (Figure 6a). In order to further verify these findings in other CSCs models, we examined relative pp38 levels in CD133+/CXCR4+ (Dual hi) and CD133-/CXCR4- (Dual lo) cells from HT29 spheroids treated with vehicle and HS06. Interestingly, Dual hi cells showed significantly lower p38 activation at baseline (Figure 6b, 6c). However, it was robustly (~100%) induced by HS06 treatment (Figure 6b, 6c). On the other hand, Dual lo cells showed significantly weak (<33%) induction of p38 following HS06 treatment, suggesting selective p38 activation in CSCs (Figure 6b, 6c). Hence, HS06's ability to regulate critical MAPK signaling selectively in CSCs might explain its preference for inhibiting CSCs.

### HS06 inhibits TCF4 signaling in a manner dependent on p38 MAPK activation

**Figure 7:**
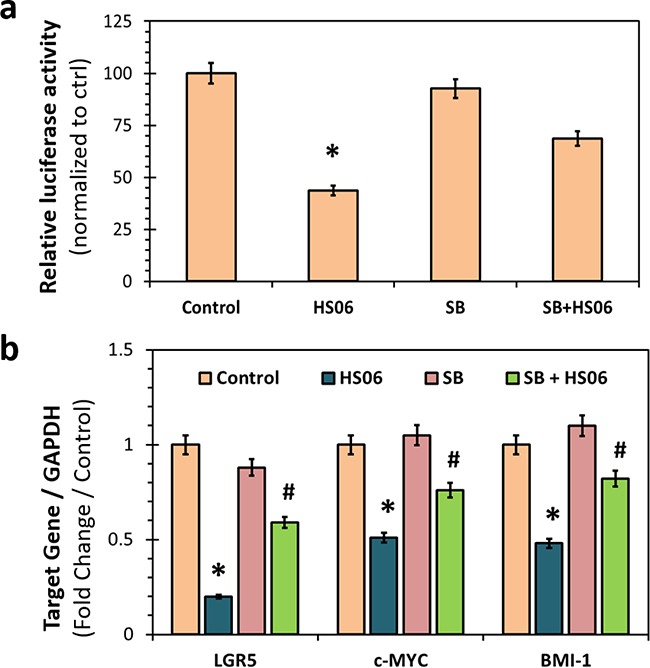
Effects of HS06 on TCF4 signaling **a**. Luciferase reporter assay showing significant inhibition of TCF/LEF promoter activity with HS06 that is reversed in the presence of p38 inhibitor – SB201350 (SB) (5 μM). **b**. Quantitative RT-PCR analysis showing inhibition of β-catenin targets gene expression (LGR5, c-MYC and BMI-1) by HS06, which is significantly reversed by SB. GAPDH was used as housekeeping control. * p-value < 0.005 compared to vehicle control. # p-value < 0.005 compared to HS06.

CSCs self-renewal and survival is likely to be regulated by one or more key developmental signaling pathways. One such pathway is TCF4 signaling, a critical regulator of colon, breast, and other CSCs’ self-renewal [30, 31]. We examined if HS06 inhibits TCF4 transcription and if so, whether p38 MAPK activation is important for such an effect. We grew HT29 cells stably transfected with TCF4 luciferase reporter as spheroids and then treated with HS06 with or without SB203580. HS06 caused a significant (50%) inhibition of TCF4 luciferase activity, which was significantly attenuated following SB203580 pre-treatment (Figure 7a). In further support, HS06 caused significant reduction in the mRNA levels of key TCF4 targets of interests i.e. CSC marker (LGR5) and self-renewal factors (c-MYC, BMI-1) which were reversed upon SB203580 pre-treatment (Figure 7b). Hence, HS06 mechanism of action involves inhibition of TCF4 transcription, which occurs at least in part through activation of p38α MAPK.

## DISCUSSION

Using a robust chemical biology approach, we have discovered phenomenal specificity exhibited by HS of distinct chain length at the levels of a) the cancer cell and its b) signal transduction machinery. The natural, most common hexasaccharide sequence of HS, but not other longer or shorter sequences, has a unique ability to induce selective and specific activation of MAPK to inhibit CSCs. The specificity of the signaling is evident by differential activation of MAPK family members in an isoform-specific manner. In particular, HS06 augments activation of p38α and attenuates activation of p38δ, without activating other MAPK family members, to induce selective inhibition of CSCs. The sum of this pleotropic signaling is likely required to effect CSC inhibition. At the cellular level, HS06 exhibits preference for CSCs as evidenced by selective inhibition of growth as well as induction of specific MAPK signaling. Importantly, selective activation of p38 MAPK was demonstrated in two different models of CSCs- spheroids and Dual hi cells that are endowed with self-renewal and other CSC like properties [32]. Because the above molecular processes are fundamental to growth and differentiation [28, 29], HS06 is likely to inhibit CSCs from many types of tissues. In fact, in our own study, several different cancer spheroids irrespective of their cellular genetic aberrations were significantly inhibited by HS06.

This work presents present major new paradigms for three distinct areas including glycobiology, cancer biology and anti-cancer therapy. With respect to the glycobiology paradigm, we have discovered that in contrast to HS06, longer HS chains exhibit a progressive loss of CSC inhibition capability, which is corroborated by changes in growth and self-renewal markers as well as contrasting biochemical changes in MAPK activation. This is a major paradigm-shifting finding that HS sequences of distinct chain lengths may have unique and completely opposed interactions. It is generally expected that longer chains bind better to proteins than shorter chains resulting in better function [22, 33]. In fact, modulation of function of many proteins, e.g., chemokines [34, 35] and coagulation proteases [33, 36], has been reported to be better with longer chains. The observation here that longer chains, e.g., HS38, exhibits loss of inhibition of CSCs suggests that the traditional expectations with regard to chain lengths may have to be revised at least in the context of cancer.

At the level of cancer biology, the role of specific and selective signal transduction modulation(s) discovered here as means to regulate CSCs phenotype will have major impact on dissecting CSCs’ specific signaling. Our findings place p38 MAPK as an important regulator of CSCs self-renewal and further corroborate its increasingly recognized role as a tumor suppressor [29, 37], especially in the context of natural agent induced inhibition of CSCs and/or apoptosis [38, 39]. More importantly, for the first time, we demonstrate an opposing role of p38 isoforms, α/β and δ, in regulation of CSC self-renewal in response to HS06. Both SB230580, a selective inhibitor of p38α/β at the doses used in the study [27] as well as genetic knockdown of p38α, reversed the anti-CSC effects of HS06. In contrast, p38δ produced opposite effects. Although p38 family members have overlapping substrate specificities, some substrates are better targeted by p38α/β than p38γ/δ and vice versa [29]. Collectively, these findings can have major implication in understanding the context specific role of p38 in regulating cancer hallmarks as well as other fundamental biological processes in which it plays a key regulatory role – stress response, differentiation, immune response, etc. [29]. In fact, such a paradoxical role of p38α and p38γ in skeletal muscle differentiation has been demonstrated before [40].

While the exact downstream targets that mediate anti-CSCs effects of p38α and p38β activation in response to HS06 treatment remain to be fully elucidated, we show for the first time its ability to negatively regulate TCF4. The TCF4 signaling pathway is known to be a major regulator of CSC phenotype in colon and breast cancer and could be one of the mechanisms involved in HS06 action [41, 42]. We predict that identifying connection(s) between known or novel p38α (and/or p38β) substrates involved in regulation of wnt-β-catenin-TCF4 may reveal novel pathways involved in CSCs self-renewal. Similarly, we suggest that identification of direct cellular targets of HS06 that regulate p38 MAPK activation should provide insight into novel cancer signaling pathways and/or potential drug targets. With respect to the latter, a thorough search of ligands and receptors belonging to growth factor (GFs), cytokine (CK) and morphogen (MG) families may reveal potential novel targets. This notion is based on the established role of HS and other GAGs in regulating receptors belonging to GF/CK/MGs, which also play a key role in regulation of CSCs self-renewal [43]. Specifically, epidermal growth factor receptor (EGFR) and its family members, hepatocyte growth factor receptor (HGFR, also known as c-MET), and more recently fibroblast growth factor receptor (FGFR) are shown to be important in modulating CSC proliferation, self-renewal, and differentiation [32, 44–46]. Thus, these cell surface receptors are likely to be putative candidates for regulation by HS06.

At the level of translational research, this work also presents a major paradigmatic result. Current anti-cancer agents fail to target CSCs, a major barrier in achieving cure. Although the exact differences between bulk cancer cells and CSCs remains unknown, differences in intracellular signaling has been suspected as a key reason in maintenance of the CSC phenotype [2, 32, 44, 46]. To this end, HS06 and its analog(s) represent promising agent(s) that may offer prevention of cancer relapse. Although sulfated saccharides (ODSH, M402 and PI-88) are currently being studied in clinical trials for different types of cancers (pancreas, leukemia, prostate, melanoma, etc.), these agents most probably do not target CSCs. Also, despite the observation that HS06 was the most optimal anti-CSC molecule among GAGs evaluated in this work, further fine-tuning of its structure by variation in sulfation and/or monomer residue content may lead to a clinically viable agent. In fact, a novel therapeutic approach to completely ‘cure’ cancer may involve dual combination therapy involving shorter HS chains and agents targeting bulk cancer cells, e.g., HS06 and chemotherapy.

Overall, this work contributes to the growing definition of HS structure–function relationships, especially in the area of cancer biology, chemical biology and cell signaling. Despite years of effort in deciphering the ‘HS-elephant’ by the best of ‘six blind men’ [21], the massive diversity of HS sequences continues to thwart simplification and present new set of paradigms. This work shows that HS plays a more complex role in driving a complex pathophysiological process such as cancer, but also presents novel avenues for its resolution.

## EXPERIMENTAL PROCEDURES

### GAG oligosaccharides

Each GAG oligosaccharide, except for the pentasaccharide HS05, was purchased from Iduron (Cheshire, UK) and used as such. The oligosaccharides share a common disaccharide repeating unit commonly found in heparin/heparan sulfate, which is IdoA2S–GlcNS6S, where IdoA2S represents 2-O-sulfated iduronic acid and GlcNS6S represents N- and 6-O-sulfated glucosamine. These oligosaccharides contain unsaturation at the non-reducing end. HS05 (or Arixtra™) was purchased from Medical College of Virginia pharmacy and refers to the pentasaccharide sequence that binds antithrombin with high affinity [47].

### Cell culture and transfection

Human colorectal (HT29, HCT116, and LS174T) and pancreatic (Panc-1) cells were obtained from ATCC within 12 months of conducting experiments directly from ATCC (Manassas, VA, USA), whereas breast MDA-MB-231 cell line was a kind gift from Dr. Kolblinski (Virginia Commonwealth University, Richmond, VA). These cells were maintained in 10 cm tissue cultured treated plate (USA Scientific) as monolayer in Dulbecco's Modified Eagle Medium: Nutrient Mixture F-12 (DMEM/F-12) (Gibco, cat# 11320-033) supplemented with 10% fetal bovine serum (FBS) (Gibco, cat # 10438-026), and 1% streptomycin/ penicillin(AA) (Gibco, cat #15240-0062). The cells were passaged using trypsin containing ethylenediaminetraacetic acid (EDTA) (Gibco, cat # 25300-054) before they reached 70% confluence. HT29 cells were transfected using mammalian p38α (Dharmacon # M-003512-02-0005) and p38δ (Dharmacon # M-003591-02-0005) smart pool as well individual p38α siRNA (Dharmacon # D-003512- 15 and D-003512-19) (25nM final concentration) with dharmafect duo reagent in a 6-well plate using dharmacon siRNA transfection protocol. Cells were plated for spheroid assay 48 hr after the transfection.

### Cell proliferation assay

Cell proliferation was evaluated by MTT (3-(4,5-dimethylthiazol-2-yl)-2,5-diphenyltetrazolium bromide) cell proliferation assay, as described earlier [10]. For HT29 cell line ~2.5×10^3^ cells/100 μL/well were plated in 96-well tissue culture treated plate. After overnight incubation at 37º C vehicle (control) or GAG was added at the desired concentration and the cells were further incubated for 60 – 72 h. At the end of the incubation, 10 μL of 5 mg/mL MTT solution (Sigma # M 2128) made in phosphate buffered saline (PBS) (Gibco # 10010-023) was added to each well and incubated for minimum of 2 to 3 hr until crystals formation was observed. Following this, 150 μL of 4 mM HCl (Sigma # A451-1) in isopropanol solution was added drop wise to each well and the mixture was triturated until the crystals dissolve completely. Finally, the plate was placed on the spectrophotometer reader and read at 590 nm and growth inhibition was calculated as percent of control. Experiments were performed at least in triplicates and averaged.

### Primary (1°) colonosphere formation assay

For primary sphere formation, cells were plated in non-treated, low adhesion, 96 wells plate at the concentration of 100 cells/100 μL/well in stem cell media (SCM) that consisted of DMEM:F12:AA (Gibco # 11320-023, Gibco # 15240-0062), supplemented with 1±B27 (Gibco # 17504-044), 20 ng/mL epidermal growth factors (Sigma # E9644) and 10 ng/mL fibroblast growth factor (Sigma # 354060). After four hour of incubation, vehicle (control) or GAG at the desired concentrations were added to each well (at least in triplicates for each sample). On day five, numbers of spheres ranging from 50 – 150 mm in diameter were counted using phase contrast microscope and percent inhibition was calculated compared to control.

### Secondary (2°) and tertiary (3°) colonosphere assay

For 2° colonospheres, the 96-well plate of primary spheres was centrifuged at speed of 1000 rpm for 1 min and the supernatant was removed. Spheres that settled at the base of the plate were trypsinized with 20 μL/well and single cell suspension was prepared using vigorous mechanical dissociation. The numbers of viable cell were counted with 1:5 ratio of cell:trypan blue and then re-plated at 100 cells/100 mL/well in SCM media in a low adhesion plate. No further treatment with GAG was performed . Numbers of spheres were counted as above on day 5. The same method was repeated for 3° spheres.

### Western blotting analysis

Western blot analysis was performed according to the standard protocol described in the literature as well as in our earlier work (*9*). Briefly, HT29 cells were plated in serum-free SCM in a low adhesion 6-well plate to obtain spheroids. Mature spheroids were treated on day 4 after plating, with vehicle or GAGs for indicated time and cells were solubilized in lysis buffer (20 mM Na_3_PO_4_, 100 mM NaCl, 2 mM EDTA, 1% Nonidet P-40, 2.5 mM Na_3_VO_4_) containing protease (Roche) as well as phosphatase inhibitor cocktails (Sigma). Following centrifugation at 14,000 g for 15 min, the supernatant was used for Western blot analysis. In all analyses, protein concentration was determined by the Bio-Rad Protein Assay kit (Bio-Rad, Hercules, CA). Approximately 25–50 μg of protein was separated by polyacrylamide gel electrophoresis and was transferred to PVDF membrane (Bio-Rad, Hercules, CA). Blocking was done with 5% BSA for 1 hr followed by overnight incubation with primary antibody (dilution 1:1000): anti-CD44 (Cell Signaling #3570S), anti-EpCAM (Cell Signaling #2929S), anti-LGR5 (Origene # TA503316), anti-BMI-1 clone F6 (Monoclonal IgG1) (Millipore # 05-637), anti-c-MYC (Millipore # 06-340) and anti-CK20 (Abcam # ab76126), anti-phospho p38 (Cell Signaling #4511), anti-phospho ERK1/2 (Cell Signaling #4370), and anti-phospho JNK1/2/3 (Cell Signaling #4668, anti-p38 (Cell Signaling #8690), anti-ERK1/2 (Cell Signaling #4695), and anti- JNK1/2/3 (Cell Signaling #9252), anti-p38α (Cell Signaling #9218), anti-p38β (Cell Signaling #2339), anti-p38γ (Cell Signaling #2307), anti-p38δ (Cell Signaling #2308). This was followed by incubation with appropriate secondary antibody and protein bands were visualized using the enhanced chemiluminescence detection system and imaged with LAS-3000 Imaging System (FUJIFILM). Densitometry was determined by AIDA image analyzer software (Raytest, Germany) and results were calculated as relative intensity compared to control. All experiments were performed at least three times and averaged.

### Flow cytometry analysis

Human colon cancer HT29 cells, grown in spheroid condition and treated with vehicle or GAG for 24 hrs, were trypsinized and single cells were re-suspended at 10^6^ cells/mL in PBS buffer. Cells were incubated with LGR5-PE (anti-human mouse monoclonal MAB clone 2A2) (Dilution 1:50) (Origene, Rockville, MA) antibody for 30 min at 4 ºC and washed once with PBS buffer prior to analysis. Cell sorting was performed using FACSAria™ II High-Speed Cell Sorter (BD Biosciences, San Jose, CA) and data were analyzed with FCS Express 4 Flow Cytometry software (De-Novo Software, Los Angeles, CA).

### Differentiation assay

Differentiation assay on mature HT29 colonosphere was performed, as reported earlier (*9*). The HT29 colonospheres were treated with vehicle or 100 μM GAG for 24 hrs prior to making single cell suspension and plating them on collagen coated glass cover slips or flasks in the presence of media supplemented with 2.5% fetal bovine serum containing GAG or vehicle. At indicated time points after plating, cells lysate was examined for CK-20 expression with western-blot as described above.

### Apoptosis assay

Human colon cancer HT29 cells, grown in spheroid condition were treated with vehicle or GAGs for 24 hours. Following which cells were trypsinized and single cells were re-suspended at 10^6^ cells/mL in PBS buffer. Two different methods were used to assess apoptosis induction. In the first methods, cells were incubated with propidium iodide and Annexin V-APC (#88-8007 ebioscience, San Diego, CA) and flow cytometric analyses were performed as above. In the second method, fluorescence microscopy was employed to examine morphological changes suggestive of apoptosis following staining with 1:1 mixture of 100 μg/ml each of acridine orange (AO) and ethidium bromide (EB) prepared in PBS (Spector DL, *et al*.). Briefly, a small volume of cell suspension was mounted on a glass slide and incubated with 1 μl of AO/EB solution and mixed gently just prior to microscopy and quantification. At least 500 cells in 10 – 15 fields were examined in each sample using Nikon ECLIPSE E800M fluorescence microscope using 20X objective. Results were quantitated as proportion of cells exhibiting characteristic apoptotic morphology normalized to vehicle treated controls. The data was expressed as apoptosis index = [([apoptotic cells (GAGs)/total cells (GAGs)] / [apoptotic cells (vehicle)/total cells (vehicle)]).

### Real-time reverse transcriptase polymerase chain reaction (QPCR)

Total RNA was isolated using Trizol kit (Ambion by Life technologies Inc.) 1 μg total RNA was reverse transcribed using First-Strand cDNA synthesis Kit using 10X primer mix (Cat # 75780, Affymetrix) as per manufacturer protocol. QPCR was performed using VeriQuest Fast SYBR Green qPCR Master Mix (Affymetrix) in a 7500 fast real time machine (Applied Biosystem). Relative expressions of mRNA were calculated using ΔΔCT methods using GAPDH a control. Following primers were used in the current study: Forward primer LGR5 – 5’ CTC CCA GGT CTG GTG TGT TG 3’; Reverse primer LGR5 – 5’ GAG GTC TAG GTA GGA GGT GAA G 3’; Forward primer c-MYC - 5’ GGC TCC TGG CAA AAG GTC 3’; Reverse primer c-MYC - 5’ AGT TGT GCT GAT GTG TGG AGA 3’; Forward primer BMI-1 - 5’ GGC TCC TGG CAA AAG GTC 3’; Reverse primer BMI-1 - 5’ AGT TGT GCT GAT GTG TGG AGA 3’; Forward Primer GAPDH- 5’ TGT TGC CAT CAA TGA CCC CTT- 3’; Reverse Primer GAPDH- 5’CTC CAC GAC GTA CTC AGC G - 3’

### Luciferase reporter assay

HT29 cells stably expressing TCF/LEF reporter and Renilla reporter has been generated in our lab. Briefly, HT29 cells, grown as colonosphere were treated with HS06 (100uM) alone or combination with p38 inhibitor SB201350 (SB) for 24 hr. Luciferase activity was measured on Titertek Berthold single tube luminometer by using the Dual Luciferase reporter assay system (Promega, Madison, WI) according to the manufacturer's protocol and data was normalized with renilla signal control and plotted.

### aPTT assay

Clotting time was measured in a standard one-stage recalcification assay with a BBL Fibrosystem fibrometer (Becton− Dickinson, Sparles, MD). In the APTT assay, x μL of oligosaccharides HSO5, HSO6, HSO8 and UFH were mixed with (100-x) μL of citrated human plasma and 100 μL of prewarmed APTT reagent (0.2% ellagic acid). After incubation for 4 min at 37 °C, clotting was initiated by adding 100 μL of prewarmed 25 mM CaCl2, and the time to clot was noted. The data were fitted to a quadratic trend line, which was used to determine the concentration of the oligosaccharides necessary to double the clotting time. Clotting time in the absence of an anticoagulant was determined in a similar fashion using 10 μL of deionized water and/or the appropriate organic vehicle and was found to be 33.5 s on an average for APTT.

### Phospho kinase array

HT29 cells were grown as spheres in 6-well non-treated plates (low adhesion). Treatment with vehicle, HS06, or HS38 at 100 μM concentration was added on day four and lysate was prepared at 15 min according to the manufacturer's protocol Human Phospho-Kinase Array (ARY001; R&D, Minneapolis, MN) kit. Bright signal was observed for each of the positive controls on the membrane confirming the validity of the results and densitometry was done using AIDA software.

### Statistical analysis

All data are expressed as means ±SEM unless otherwise indicated. The results were analyzed using the unpaired, two-tailed Student's *t*-test. *P* <0.01 was designated as the level of significance unless specified otherwise.

## SUPPLEMENTARY MATERIALS FIGURES AND TABLES



## References

[1] Pattabiraman DR, Weinberg RA. (2014). Tackling the cancer stem cells - what challenges do they pose?. Nature reviews Drug discovery.

[2] Clevers H. (2011). The cancer stem cell: premises, promises and challenges. Nature medicine.

[3] O’Brien CA, Pollett A, Gallinger S, Dick JE. (2007). A human colon cancer cell capable of initiating tumour growth in immunodeficient mice. Nature.

[4] Ricci-Vitiani L, Lombardi DG, Pilozzi E, Biffoni M, Todaro M, Peschle C, De Maria R. (2007). Identification and expansion of human colon-cancer-initiating cells. Nature.

[5] Reya T, Morrison SJ, Clarke MF, Weissman IL. (2001). Stem cells, cancer, and cancer stem cells. Nature.

[6] Ye X, Weinberg RA. (2015). Epithelial-Mesenchymal Plasticity: A Central Regulator of Cancer Progression. Trends in cell biology.

[7] Kleffel S, Schatton T. (2013). Tumor dormancy and cancer stem cells: two sides of the same coin?. Advances in experimental medicine and biology.

[8] Jin L, Hope KJ, Zhai Q, Smadja-Joffe F, Dick JE. (2006). Targeting of CD44 eradicates human acute myeloid leukemic stem cells. Nature medicine.

[9] Li Y, Rogoff HA, Keates S, Gao Y, Murikipudi S, Mikule K, Leggett D, Li W, Pardee AB, Li CJ. (2015). Suppression of cancer relapse and metastasis by inhibiting cancer stemness. Proceedings of the National Academy of Sciences of the United States of America.

[10] Patel NJ, Karuturi R, Al-Horani RA, Baranwal S, Patel J, Desai UR, Patel BB. (2014). Synthetic, non-saccharide, glycosaminoglycan mimetics selectively target colon cancer stem cells. ACS chemical biology.

[11] Hanahan D, Weinberg RA. (2011). Hallmarks of cancer: the next generation. Cell.

[12] Ibrahim SA, Hassan H, Gotte M. (2014). MicroRNA regulation of proteoglycan function in cancer. The FEBS journal.

[13] Yip GW, Smollich M, Gotte M. (2006). Therapeutic value of glycosaminoglycans in cancer. Molecular cancer therapeutics.

[14] Couchman JR., Schekman R, Goldstein L, Lehmann R ((2010)). Transmembrane Signaling Proteoglycans. Annual Review of Cell and Developmental Biology.

[15] Nikitovic D, Chatzinikolaou G, Tsiaoussis J, Tsatsakis A, Karamanos NK, Tzanakakis GN. (2012). Insights into Targeting Colon Cancer Cell Fate at the Level of Proteoglycans/Glycosaminoglycans. Current medicinal chemistry.

[16] Sergeant N, Lyon M, Rudland PS, Fernig DG, Delehedde M. (2000). Stimulation of DNA synthesis and cell proliferation of human mammary myoepithelial-like cells by hepatocyte growth factor/scatter factor depends on heparan sulfate proteoglycans and sustained phosphorylation of mitogen-activated protein kinases p42/44. Journal of Biological Chemistry.

[17] Rolny C, Spillmann D, Lindahl U, Claesson-Welsh L. (2002). Heparin amplifies platelet-derived growth factor (PDGF)-BB-induced PDGF alpha-receptor but not PDGF beta-receptor tyrosine phosphorylation in heparan sulfate-deficient cells - Effects on signal transduction and biological responses. Journal of Biological Chemistry.

[18] Bishop JR, Schuksz M, Esko JD. (2007). Heparan sulphate proteoglycans fine-tune mammalian physiology. Nature.

[19] Sarkar A, Desai UR. (2015). A Simple Method for Discovering Druggable, Specific Glycosaminoglycan-Protein Systems. Elucidation of Key Principles from Heparin/Heparan Sulfate-Binding Proteins. PloS one.

[20] Patel N, Baranwal S, Patel BB. (2015). A strategic approach to identification of selective inhibitors of cancer stem cells. Methods in molecular biology.

[21] Varki A. Six blind men and the elephant--the many faces of heparan sulfate. Proceedings of the National Academy of Sciences of the United States of America.

[22] Joseph PR, Mosier PD, Desai UR, Rajarathnam K. (2015). Solution NMR characterization of chemokine CXCL8/IL-8 monomer and dimer binding to glycosaminoglycans: structural plasticity mediates differential binding interactions. The Biochemical journal.

[23] Pedron S, Kasko AM, Peinado C, Anseth KS. (2010). Effect of heparin oligomer chain length on the activation of valvular interstitial cells. Biomacromolecules.

[24] Cancer Genome Atlas N (2012). Comprehensive molecular characterization of human colon and rectal cancer. Nature.

[25] Vaiopoulos AG, Kostakis ID, Koutsilieris M, Papavassiliou AG. (2012). Colorectal cancer stem cells. Stem cells.

[26] O’Brien CA, Kreso A, Jamieson CH. (2010). Cancer stem cells and self-renewal. Clinical cancer research : an official journal of the American Association for Cancer Research.

[27] Cohen P. (1997). The search for physiological substrates of MAP and SAP kinases in mammalian cells. Trends in cell biology.

[28] Cuadrado A, Nebreda AR. (2010). Mechanisms and functions of p38 MAPK signalling. The Biochemical journal.

[29] Cuenda A, Rousseau S. (2007). p38 MAP-kinases pathway regulation, function and role in human diseases. Biochimica et biophysica acta.

[30] Qiao L, Xu ZL, Zhao TJ, Ye LH, Zhang XD. (2008). Dkk-1 secreted by mesenchymal stem cells inhibits growth of breast cancer cells via depression of Wnt signalling. Cancer letters.

[31] Reya T, Clevers H. (2005). Wnt signalling in stem cells and cancer. Nature.

[32] Sharon C, Baranwal S, Patel NJ, Rodriguez-Agudo D, Pandak WM, Majumdar AP, Krystal G, Patel BB. (2015). Inhibition of insulin-like growth factor receptor/AKT/mammalian target of rapamycin axis targets colorectal cancer stem cells by attenuating mevalonate-isoprenoid pathway in vitro and in vivo. Oncotarget.

[33] Olson ST, Halvorson HR, Bjork I. (1991). Quantitative characterization of the thrombin-heparin interaction. Discrimination between specific and nonspecific binding models. The Journal of biological chemistry.

[34] Raghuraman A, Mosier PD, Desai UR. (2006). Finding a needle in a haystack: development of a combinatorial virtual screening approach for identifying high specificity heparin/heparan sulfate sequence(s). Journal of medicinal chemistry.

[35] Sankaranarayanan NV, Desai UR. (2014). Toward a robust computational screening strategy for identifying glycosaminoglycan sequences that display high specificity for target proteins. Glycobiology.

[36] Olson ST, Swanson R, Raub-Segall E, Bedsted T, Sadri M, Petitou M, Herault JP, Herbert JM, Bjork I. (2004). Accelerating ability of synthetic oligosaccharides on antithrombin inhibition of proteinases of the clotting and fibrinolytic systems. Comparison with heparin and low-molecular-weight heparin. Thrombosis and haemostasis.

[37] Bradham C, McClay DR. (2006). p38 MAPK in development and cancer. Cell cycle.

[38] Yang Y, Li Y, Wang K, Wang Y, Yin W, Li L. (2013). P38/NF-kappaB/snail pathway is involved in caffeic acid-induced inhibition of cancer stem cells-like properties and migratory capacity in malignant human keratinocyte. PloS one.

[39] Pollock CB, McDonough S, Wang VS, Lee H, Ringer L, Li X, Prandi C, Lee RJ, Feldman AS, Koltai H, Kapulnik Y, Rodriguez OC, Schlegel R (2014). Strigolactone analogues induce apoptosis through activation of p38 and the stress response pathway in cancer cell lines and in conditionally reprogrammed primary prostate cancer cells. Oncotarget.

[40] Lluis F, Perdiguero E, Nebreda AR, Munoz-Canoves P. (2006). Regulation of skeletal muscle gene expression by p38 MAP kinases. Trends in cell biology.

[41] Korkaya H, Paulson A, Charafe-Jauffret E, Ginestier C, Brown M, Dutcher J, Clouthier SG, Wicha MS. (2009). Regulation of mammary stem/progenitor cells by PTEN/Akt/beta-catenin signaling. PLoS biology.

[42] Hoffmeyer K, Raggioli A, Rudloff S, Anton R, Hierholzer A, I Del Valle, Hein K, Vogt R, Kemler R. (2012). Wnt/beta-catenin signaling regulates telomerase in stem cells and cancer cells. Science.

[43] Sasisekharan R, Shriver Z, Venkataraman G, Narayanasami U. (2002). Roles of heparan-sulphate glycosaminoglycans in cancer. Nature reviews Cancer.

[44] Herreros-Villanueva M, Zubia-Olascoaga A, Bujanda L. (2012). c-Met in pancreatic cancer stem cells: therapeutic implications. World journal of gastroenterology : WJG.

[45] Knelson EH, Gaviglio AL, Nee JC, Starr MD, Nixon AB, Marcus SG, Blobe GC. (2014). Stromal heparan sulfate differentiates neuroblasts to suppress neuroblastoma growth. The Journal of clinical investigation.

[46] Nautiyal J, Du J, Yu Y, Kanwar SS, Levi E, Majumdar AP. (2012). EGFR regulation of colon cancer stem-like cells during aging and in response to the colonic carcinogen dimethylhydrazine. American journal of physiology Gastrointestinal and liver physiology.

[47] Atha DH, Lormeau JC, Petitou M, Rosenberg RD, Choay J. (1987). Contribution of 3-O- and 6-O-sulfated glucosamine residues in the heparin-induced conformational change in antithrombin III. Biochemistry.

