# Supplementary material for: Heparan sulfate hexasaccharide selectively inhibits cancer stem cells self-renewal by activating p38 MAP kinase
Source: Oncotarget. 2016 Sep 30;7(51):84608–22. doi: 10.18632/oncotarget.12358 (PMC5356685; doi:10.18632/oncotarget.12358)
Supplement: Supplementary file 1 [file oncotarget-07-84608-s001.pdf]

Heparan sulfate hexasaccharide selectively inhibits cancer stem cells self-renewal by activating p38 MAP kinase

SUPPLEMENTARY FIGURES AND TABLES

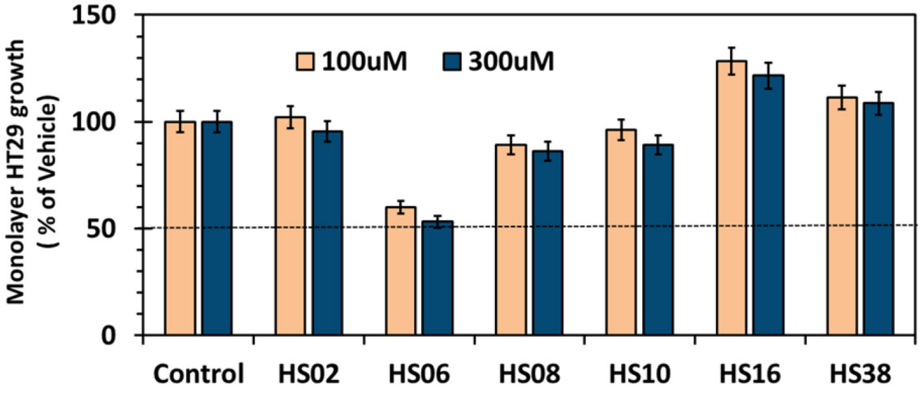

Supplementary Figure S1: Primary (1°) screening of the growth of colorectal HT29 cells under monolayer conditions in the presence of sulfated GAGs. Screening revealed lack of significant growth inhibition by sulfated GAGs in CSC-poor monolayer condition, as measured by MTT assay (OD@590 nM). HT29 cells were grown in 1% serum containing growth media under adherent condition and treated with GAGs (100 & 300 μM) for 72 hr. Cell growth in the absence of GAGs was used as 100% growth control. Data is shown as % of vehicle control. Error bars represent ±1 SEM.

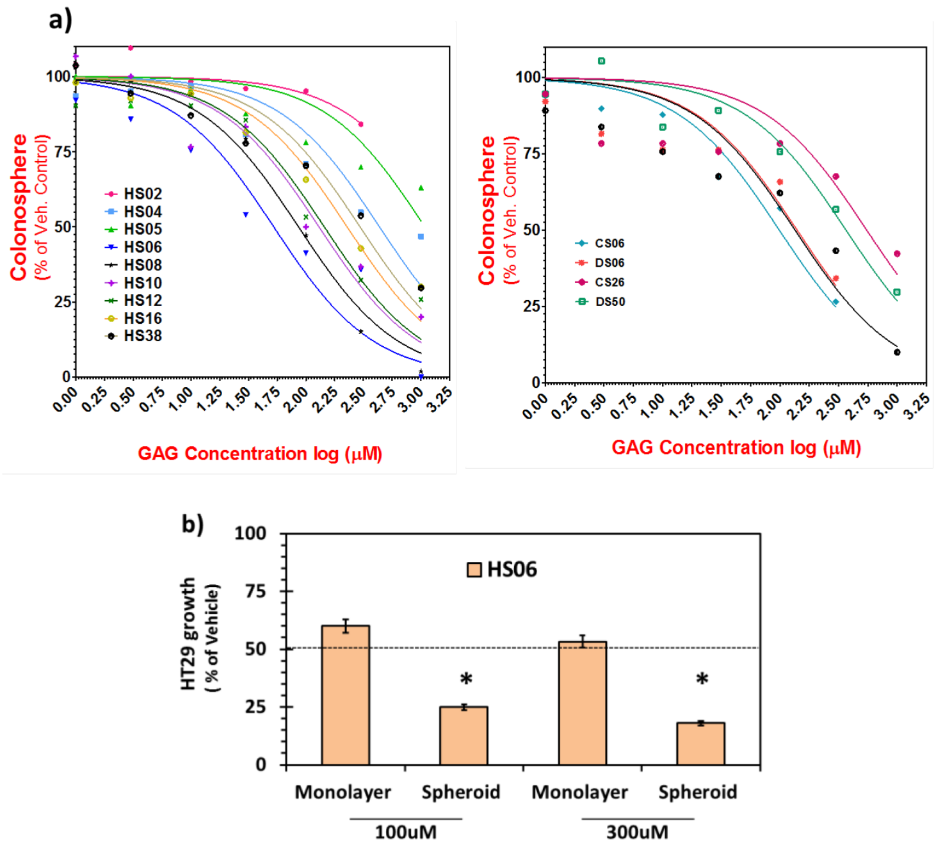

Supplementary Figure S2: a. Dose–response profile of HT29 1° spheroid (CSCs) growth in the presence of sulfated GAGs. Many GAGs showed dose-dependent inhibition of CSC growth. The cells were grown in CSC media under non-adherent conditions in the presence of GAGs (0→500 µM) for 72 hr. **b.** HS06 shows significantly greater growth inhibition in spheroid compared to monolayer condition in a dose-dependent fashion. HS = heparan sulfate; CS = chondroitin sulfate; DS = dermatan sulfate. Data is normalized to vehicle control. Error bars represent ±1 SEM. * p-value < 0.005.

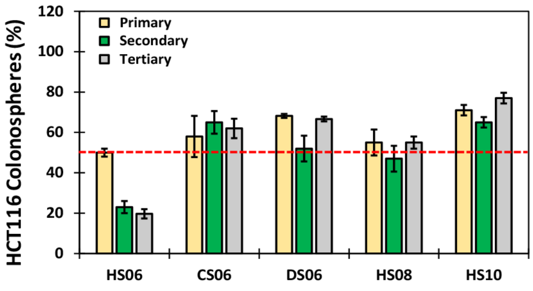

Supplementary Figure S3: Primary (1°), secondary (2°) and tertiary (3°) screening of the growth of colorectal HCT116 cells under spheroid conditions. 1 screening was performed in the presence of 100 µM sulfated GAG, whereas 2° and 3° spheroid growth was performed in the absence of further GAG treatment in HCT116 cells (p53 wild type, K-RAS mutant, microsatellite stable). HS06 was the only GAG that satisfied the predefined cutoff of >50% inhibition in both 2° and 3° spheroids growth. Data is presented as percent of the vehicle control. Error bars represent ±1 SEM.

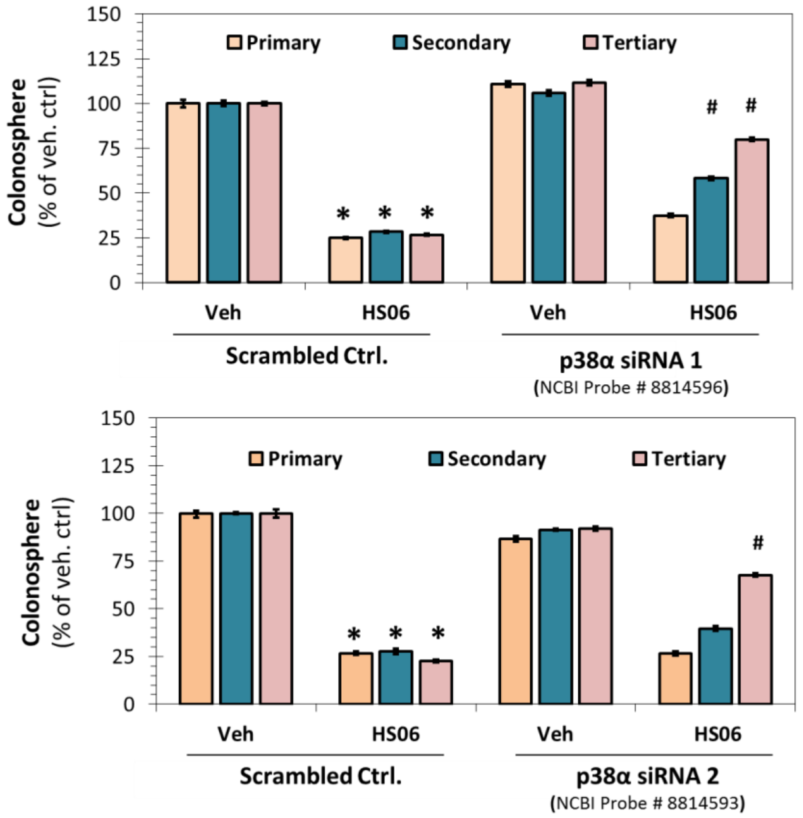

Supplementary Figure S4: Effect of p38α knockdown on HS06 mediated inhibition of CSCs self-renewal. Primary (1°), secondary (2°) and tertiary (3°) spheroid growth of colorectal HT29 cells following transfection with specific p38α siRNA sequences (NCBI probe IDs-8814596 and 8814593) following treatment with HS06. Appropriate vehicle treatment and scrambled transfection controls were used in the above experiments.

Supplementary Table S1: Comparative anticoagulant and anti-CSC properties of HSGAGs

	HSGAG	2X aPTT (μM)	IC50 sphere formation (μM)
**Anticoagulant**	**HS05**	**34**	**1078**
	**HS16**	**0.055**	**227**
**Non-anticoagulant**	**HS06**	**600**	**52**
	**HS08**	**84**	**89**

Supplementary Table S2: Effects of HS06 on activation of various phosphokinase (Human phosphokinase array)

No.	Phosphokinase	Relative densitometry value of the phosphorylated protein compared to vehicle control
1	p38α	189
2	Chk-2	170
3	p70 S6 Kinase	148
4	PRAS40	146
5	ERK1/2	138
6	MSK1/2	128
7	p70 S6 Kinase	122
8	JNK1/2/3	121
